# Evaluation of Bone Response to a Nano HA Implant Surface on Sinus Lifting Procedures: Study in Rabbits

**DOI:** 10.3390/jfb13030122

**Published:** 2022-08-21

**Authors:** Sergio H. L. Martins, Uislen B. Cadore, Arthur B. Novaes, Michel R. Messora, Bruna Ghiraldini, Fabio J. B. Bezerra, Daniele Botticelli, Sergio L. S. de Souza

**Affiliations:** 1 Department of Oral and Maxillofacial Surgery and Periodontology, School of Dentistry of Ribeirao Preto, University of Sao Paulo, Ribeirao Preto 14040-904, SP, Brazil; 2 ARDEC (Ariminum Research & Dental Education Center) Academy, Viale Giovanni Pascoli 67, 47923 Rimini, Italy

**Keywords:** animal experiments, biomaterials, bone implant interactions, bone substitutes, guided bone regeneration, sinus floor elevation

## Abstract

The aim of this study was to evaluate the bone response to two different implant surfaces on sinus lift procedures in rabbits. Bilateral sinus lifting with inorganic bovine bone associated with collagen membrane and immediate implantation were performed in 16 rabbits. Custom mini-implants were randomly installed in the prepared sites: one side received a double acid-etched (DAE) surface and the other a nano-hydroxyapatite (NHA) surface. The animals were euthanized 30 and 60 days after surgery, and biopsies were collected for microtomographic and histomorphometric analysis. After 30 days, no intra- and inter-group statistical differences were observed in microtomographic analysis, while at 60 days, bone analysis showed statistically significant differences between groups (*p* < 0.05) for all the evaluated parameters. Histomorphometric analysis showed, after 30 days, mean % of Bone-to-Implant Contact (BIC) for DAE and NHA of 31.70 ± 10.42% vs. 40.60 ± 10.22% (*p* > 0.05), respectively; for % of Bone Area Fraction Occupancy (BAFO), mean values were 45.43 ± 3.597% for DAE and 57.04 ± 5.537% for NHA (*p* < 0.05). After 60 days, mean %BIC and %BAFO for DAE and NHA implants were statistically significant (*p* < 0.05). The NHA surface showed superior biological features compared to the DAE treatment, promoting higher bone formation around the implants in an experimental model of bone repair in a grafted area.

## 1. Introduction

Advances in the implant characteristics have been proposed over the years to improve the process of osseointegration, especially in challenging biological situations [1]. It includes changes in macrostructure [2], addition of biological compounds [3], and microstructure surface [4,5].

Both the morphology and the surface roughness of the implants have an important influence on cell proliferation, differentiation, and in the synthesis of the extracellular matrix [6,7]. Roughness facilitates retention of osteogenic cells and allows migration to the implant surface through osseoconduction [8], promoting better collagen adherence and increasing the surface area, the number of sites for cell fixation, leading to greater tissue growth and mechanical stability [9,10,11].

Implant surfaces evolved from smooth to moderately rough, with some chemical changes [12,13]. Roughening implant surface increases osteoconductivity, and the biological response seems to differ according to different nanostructures [14,15]. Nanostructures smaller than 100 nm are more effective in cell integration [16] and have demonstrated more bone cells to attach and proliferate [17]. Nanometric structures formed with nano-hydroxyapatite deposition demonstrated a possible bioactivity in preclinical animal studies with an early increase in bone formation around implants [18]. This bioactivity could be of use on poor bone implantation sites or when the use of bone substitutes is needed, such as sinus lifting procedures.

The DAA surface is well established in the literature and produces a surface with medium roughness on the micrometer scale [19]. Hydroxyapatite acts as a topographic agent; nanoscale structures help osteoblasts to synthesize mineral matrix [20]. NHA also acts as a chemical agent that optimizes the osseointegration process [21]. This action on bone mineralization may result from soluble HA deposited on the surface and it is very similar to that found in bone, with low crystallinity and small particles, being partially metabolized by the bone. The result of this treatment generates a surface with hydrophilic characteristics [22,23]. A previous study in rats [24] found an early increase in bone formation in implants modified with nanoHA, which according to the authors may have been a result of the possible chemical bioactivity of HA or the topography of the implemented nanostructures. Another recent study [25], addressing gene expression around nanoHA-coated implants, observed that there was a significant increase in osteogenic gene expression, suggesting that nanoHA is actively involved in bone formation.

The objective of the maxillary sinus graft procedure is to obtain bone quantity and quality that allow the insertion of osseointegrated implants. Bone formation can be accelerated by inserting a biocompatible scaffold, thereby allowing bone growth through a process called osteoconduction [26]. In this process, the floor of the maxillary sinus plays an important role, as it functions as a source of cells for bone formation [27].

Studies have shown that when no biomaterial is used to elevate the floor of the maxillary sinus, bone gain is limited and the apex of the implant is surrounded by non-osseointegrated connective tissue [28,29,30]. A systematic review evaluated the effect of elevating the maxillary sinus without using graft material [31]. The average gain in height of the residual crestal bone was only 3.43 ± 0.09 mm.

The sinus lifting surgery may be difficult to perform, but the contained environment for the graft material within the sinus may provide a favorable outcome [32]. The grafted biomaterial can be well retained by the bone walls and the sinus membrane and, therefore, can be stabilized without the aid of a fixation device during the healing phase [33]. The osteogenic potential is promoted mainly by the surrounding bony walls. In addition, the Schneider membrane also has an osteogenic effect [34].

Thus, taking into account the development of new implant surfaces aimed at qualitative and quantitative improvement of the osseointegration, and on the other hand the challenge in maximizing bone-implant contact in areas of bone regeneration with the use of biomaterials, it would be interesting to carry out an in vivo study, comprising healthy rabbits submitted to maxillary sinus lifting procedures, to evaluate an implant surface modified by the addition of nano-hydroxyapatite.

## 2. Materials and Methods

The present research project was approved by the Ethics Committee on Animal Experimentation of the School of Dentistry of Ribeirao Preto-USP (protocol number 2017.1.315.58.1). The procedures were carried out in accordance with the ethical rules governed by the Brazilian College of Animal Experimentation (COBEA) and the Animal Research: Reporting In Vivo Experiments (ARRIVE) guidelines checklist.

### 2.1. Sample Size Calculation

Based on Yoon et al. [32], the sample size was determined to provide 80% power, in order to recognize a significant difference of 13.47% between groups with a 95% confidence interval (alpha = 0.05) and intragroup standard deviation of 18.03%, considering the changes in Bone Volume Fraction (BV/TV) as the primary outcome variable. A sample size of six rabbits were needed in the present study. Assuming a dropout rate of 20%, each experimental group contained eight rabbits.

Sixteen male adult New Zealand rabbits, weighing 2.5 kg to 3.2 kg, aged six months, were selected for the study. The animals were kept in appropriate cages with food and water ad libitum before and during the experimental period and remained in the Vivarium of the Faculty of Dentistry of Ribeirao Preto, FORP-USP in an environment with a cycle of 12 h of light, and temperature between 22 and 24 °C.

### 2.2. Implant Surfaces Preparation

The implants’ surfaces were prepared by SIN Implant System (São Paulo, SP, Brazil), the manufacturer, according to the sequence previously published [25]. Briefly, the DAE surface implants were produced in CNC lathe machines, from commercially pure titanium (Grade 4) cylindrical bars. After that, they received an automated pre-washing and hygiene process, carried out inside controlled rooms (Clean Room). In sequence, the implant surface received baths of nitric acid followed by sulfuric acid, in a micro corrosion process. For the NANO surface treatment, a DAE implant surface was processed: coating liquid containing nanohydroxyapatite crystals was applied on top of the implant to be coated. The HA crystals are fully synthetic, the crystals are made to precipitate from water soluble calcium and phosphate salts, directly in the coating solution. The implant was placed on a spin coater device. The implant was rotated at 2600 rpm for 3 s, for homogenization of the liquid over the entire surface, and allowed to dry at 10 min in room temperature. The implant was then placed in an oven at 450 °C for 5 min, for sintering and stable adhesion of the HA crystals.

### 2.3. Sinus Lift and Installation of Implants

**Figure 1:**
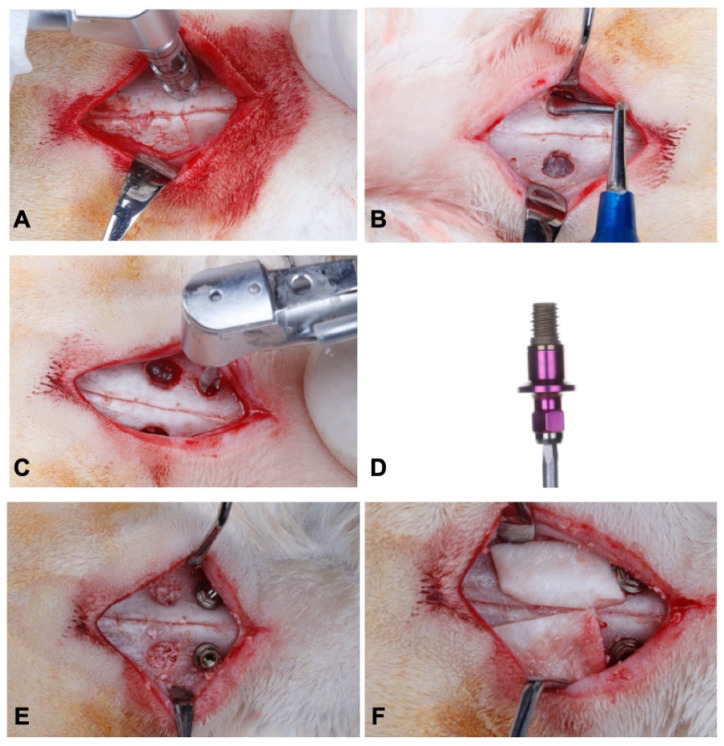
Surgical procedures: (**A**) Osteotomy with 5.0 mm trephine; (**B**) Sinus membrane elevation; (**C**) Drilling for implant insertion; (**D**) micro implant 3.0 × 4.0 mm (SIN Implant System); (**E**) Inorganic bovine bone graft in small granules (Geistlich Bio-Oss^®^) filling the maxillary sinus; (**F**) Placement of resorbable collagen membrane (Geistlich Bio-Gide^®^) on the maxillary sinus window.

The study followed a split mouth design. Implants were installed in accordance to a previously described protocol [32,35]. General anesthesia was induced by the association of Ketamine Hydrochloride (Agener Uniao Ltd., Sao Paulo, SP, Brazil), Xylazine Hydrochloride (Rompum; Bayer SA, Sao Paulo, SP, Brazil), and acepromazine (Univet, São Paulo, Brazil), in doses of 35 mg/kg, 5 mg/kg, and 0.75 mg/kg, respectively, intramuscularly. A trichotomy was performed on the nasal dorsum, then local asepsis, using a 1% PVPI solution. Subsequently, local anesthesia with 2% lidocaine and 1:100,000 adrenaline was also administered. An incision in the skin along the midline of the nasal bone was made in order to expose the dorsal surface. Two circular windows, with a diameter of 5 mm, were prepared on both sides of the nasal bone using a trephine drill (S.I.N.—Implant System, Sao Paulo, Brazil), under abundant saline irrigation (Figure 1A). The sinus mucosa was carefully elevated, and the implant installation sites were prepared 3 mm anterior to the bone windows (Figure 1B).

After the mucosa elevation of the two maxillary sinuses, osteotomy was conducted to install the implants, using a progressive sequence of drills under constant irrigation with saline solution, as recommended by the manufacturer (SIN—Implant System, Sao Paulo, Brazil) (Figure 1C). After that, the implants (SIN—Implant System, Sao Paulo, Brazil), specially designed for the research and measuring 3 mm in diameter by 4 mm in length (Figure 1D), were manually installed until the implant shoulder was at the level of the cortical bone. In one of the maxillary sinuses, an implant with a double acid-etched surface treatment was installed (Control Group—CG), and in the other with a surface covered by nano-hydroxyapatite (Test Group—TG). The choice of sides was randomized using a table generated by the website Randomization.com (http://www.randomization.com, accessed on 14 February 2018).

After installing the implants, the maxillary sinuses were carefully filled with inorganic bovine bone graft in small granules (Geistlich Bio-Oss^®^ Small, Geistlich Pharma AG, Wolhusen, Switzerland) (Figure 1E). After that, each surgical site was covered with an absorbable collagen membrane (Geistlich Bio-Gide^®^, Geistlich Pharma AG, Wolhusen, Switzerland) (Figure 1F). The surgery was concluded with the primary closure of the tissues using absorbable sutures (Vicryl Ethicon 5.0, Johnson Prod., Sao Jose dos Campos, Brazil).

After surgery, the animals received a single dose of antibiotic (Penicillin G-benzathine at a dose of 0.01 mL for each 100 g of the rabbit’s body weight, Small Veterinary Pentabiotic, Fort Dodge^®^, Campinas, Brazil) and an anti-inflammatory drug (Buprenorphine, Merial, Lyon, France) at a concentration of 0.3 mg/mL, in a dose of 0.05 mg/kg each 12 h, for 3 days. The animals had no movement or feeding restrictions after surgery and were kept in appropriate cages throughout the experimental period.

The rabbits were euthanized with a dose of 100 mg/kg of 2.5% sodium thiopentate, intravenously (Thiopentax, Cristalia, Itapira, Brazil), in the periods of 30 and 60 days after the implant installation (8 animals for each time). The maxillary sinuses were removed and fixed for subsequent three-dimensional analysis by MicroCT and histological processing/histomorphometric analysis.

### 2.4. Microtomographic Analysis

**Figure 2:**
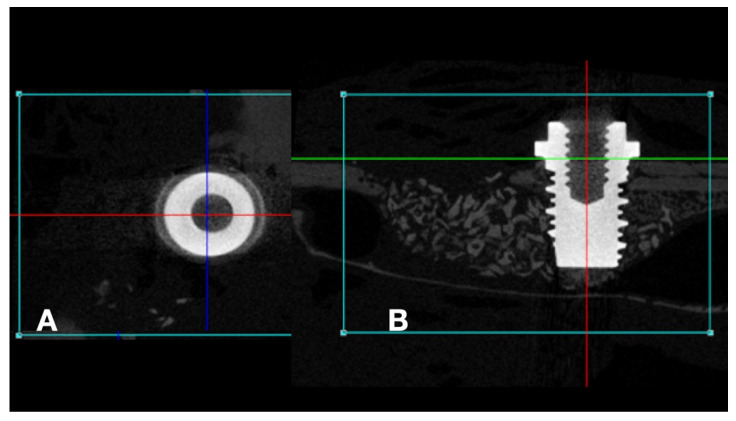
Microtomographic images: (**A**) (axial) and (**B**) (sagital). (**A**): Red line = anterior-posterior cut, Blue Line = latero-lateral cut; (**B**): Green line = axial cut, Red line = latero-lateral cut.

After 48 h of fixation in 10% buffered formaldehyde, the samples were scanned using the high resolution SkyScan 1172-160 micro-CT microtomograph (Bruker, Kontich, Antwerp, Belgium) to obtain two-dimensional tomographic projections and three-dimensional reconstruction. All scans were obtained at 100 kV and 100 μA, using an aluminum-copper filter to optimize the contrast, and set at 5.87 mm pixel size, 360° rotation, and a rotation step of 0.40. Two-dimensional tomographic projections and three-dimensional reconstruction were performed using the NRecon software (NRecon v.1.6.10.4, Bruker, Kontich, Antwerp, Belgium) and the implants were positioned on their long axis using the DataViewer software (v.1.5.0, Bruker, Kontich, Antwerp, Belgium), where it is possible to move and visualize the three axes (coronal, axial, and sagittal) (Figure 2). The axis of interest (sagittal) was selected for the complete visualization of the implants.

Subsequently, the reconstructions were subjected to morphometric analysis using the CT Analyzer software (CTAn., V.1.15.4.0, Bruker, Kontich, Antwerp, Belgium), evaluating the following parameters—the internal and external to the implant threads bone: Bone-to-Implant Contact (IS/TS, %), Bone Volume Fraction (BV/TV,%), Trabecular Thickness (Tb.Th, mm), Trabecular Number (Tb.N), and Percentage of Total Porosity (Po.Tot,%). In CTAN an advanced analysis tool (custom processing) was used, in which a list of tasks was created for analysis of the selected parameters. Measurements were made from 1 mm from the most coronal portion of the implant, reaching its entire length. This process involved determining the region of interest (ROI). The collective sum of all ROIs on a contiguous set of slices in the cross-sectional image was used to determine the volume of interest (VOI), representing the selected 3D volume. Binarization was obtained using the gray scale defined by a density of 35–150 for bone and 150–255 for implant. All micro-CT analyses were performed by a single examiner, blind to the experimental groups.

### 2.5. Histomorphometric Analysis

**Figure 3:**
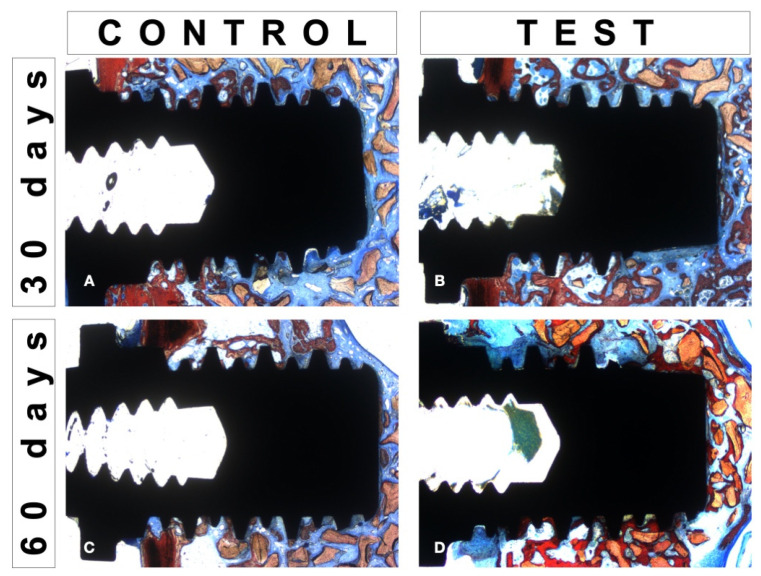
Histological images stained with Stevenel’s blue and Alizarin red for histomorphometrical evaluation: Control Group (**A**,**C**) and Test Group (**B**,**D**).

For histomorphometric evaluation (Figure 3), an image analysis software (ImageJ, NIH, Bethesda, MD, USA) was used to quantify and evaluate the osseointegration parameters around the peri-implant surface. Bone-to-Implant Contact (BIC) and Bone Area Fraction Occupancy (BAFO) were evaluated.

**Figure 4:**
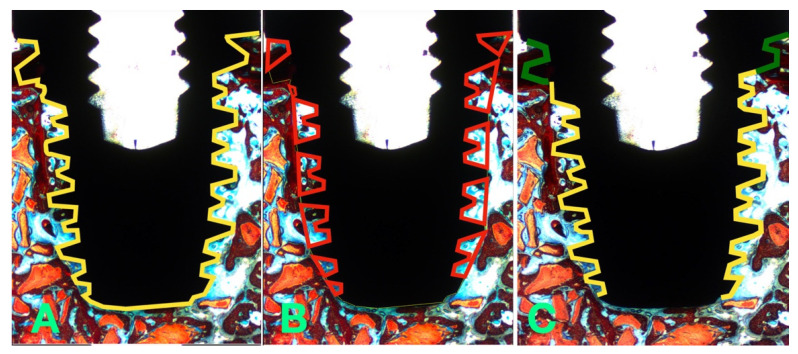
Surgical procedures—Histomorphometric Analysis: Illustrative diagram of the implant regions selected for BIC (**A**) and BAFO (**B**) measurements; (**C**) Cortical versus Grafted bone evaluation (green line—threads in contact with cortical bone/yellow line—threads in contact with grafted area).

The BIC measures the percentage of bone in contact with the perimeter of the implant surface (Figure 4A), according to the equation:

BIC = Cortical bone in contact with implant perimeter × 100 / Total implant perimeter

The BAFO measures the area of bone within the implant threads in relation to the total area delimited by it (Figure 4B) [36,37,38], according to the equation:

BAFO = Cortical bone area inside the implant threads × 100 / Total area inside the implant threads

All measurements were made by a single examiner, blind to the experimental groups.

Finally, each implant was also divided into thread portions in contact with cortical bone and thread portions in contact with grafted bone, for the histomorphometric analysis of the bone repair in the pre-existing cortical bone versus in the grafted bone (Figure 4C).

### 2.6. Statistical Analysis

Each animal was considered as the statistical unit (n = 8). The significance level was set at 5% (*p* < 0.05). The distribution of histomorphometric and microtomographic data was verified by the Shapiro–Wilk test. The data presented a normal distribution, with Student’s *t*-test being selected for the analysis of differences between groups in each period of time (30 and 60 days), while for the analysis of intra-group differences between times (30 versus 60 days), the analysis of variance (ANOVA) with Tukey’s post hoc was used. The analyses were performed with SPSS software (IBM SPSS 23, IBM Corp., Armonk, NY, USA).

## 3. Results

The healing period was uneventful. The average time of the procedures was 40.8 min in the Test Group and 41.6 min in the Control Group, without statistically significant difference between groups (*p* > 0.05).

### 3.1. Microtomographic Results

#### Inter-Group Analysis

**Figure 5:**
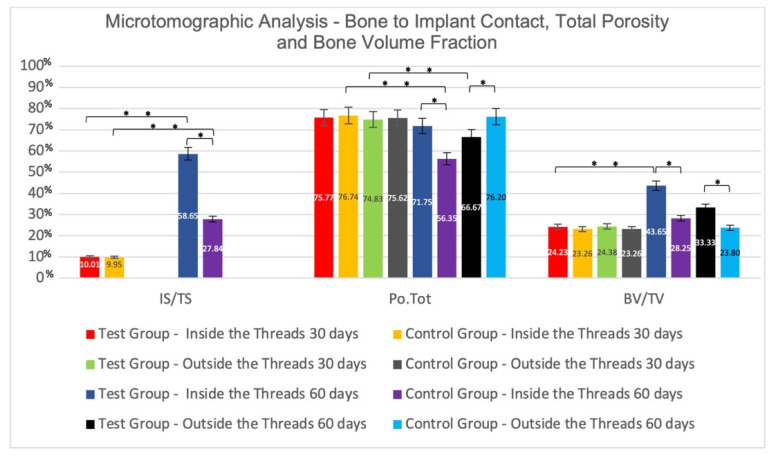
Mean values ± standard deviation of Bone-to-Implant Contact (IS/TS,%), Total Porosity (Po.Tot,%), and Bone Volume Fraction (BV/TV,%) at 30-day and 60-day microtomographic analysis. * Statistically significant difference in inter-groups comparisons (*p* < 0.05). ** Statistically significant difference in intra-groups comparisons (*p* < 0.05).

**Figure 6:**
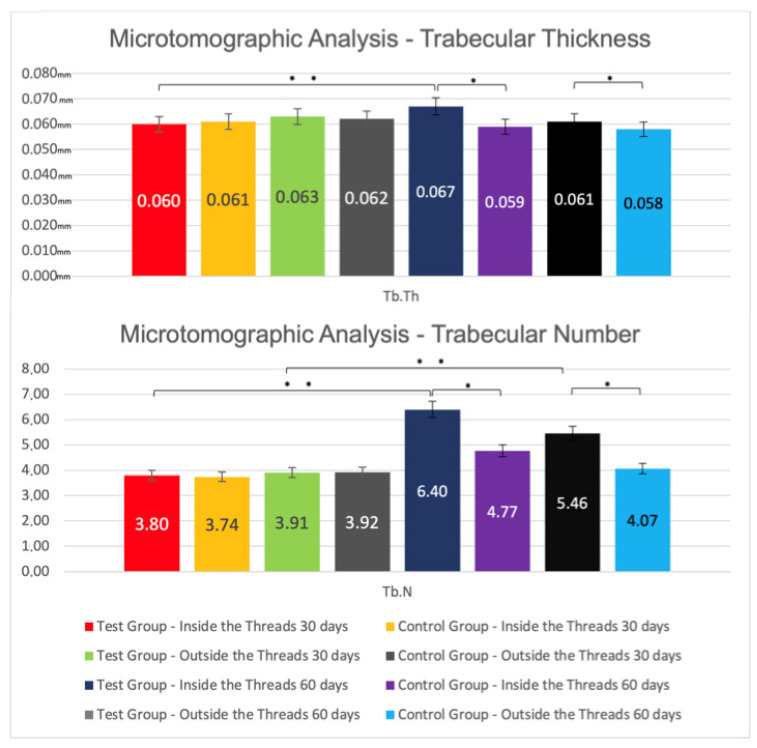
Mean values ± standard deviation of Trabecular Thickness (Tb.Th, mm) and Trabecular Number (Tb.N) at 30-day and 60-day microtomographic analysis. * Statistically significant difference in inter-groups comparisons (*p* < 0.05). ** Statistically significant difference in intra-groups comparisons (*p* < 0.05).

After 30 days, microtomographic analysis of the bone internal to implant threads showed, for IS/TS, in the Control and Test groups, mean values of 9.95 ± 0.17% vs. 10.01 ± 0.10% (*p* > 0.05), respectively. The average values of Po.Tot were, in the Test and Control groups, 75.77 ± 3.47% and 76.74 ± 3.08% (*p* > 0.05), respectively. For the BV/TV, the mean values were 24.23 ± 3.47% in the Test and 23.26 ± 3.08% in the Control Group (*p* > 0.05). For Tb.Th, there was no statistically significant difference between the Test (0.060 ± 0.004 mm) and the Control (0.061 ± 0.004 mm) groups, as well as for Tb.N (TG: 3.80 ± 0.43; CG: 3.74 ± 0.39) (*p* > 0.05). The analysis of bone external to the implant threads showed: the Po.Tot mean values were, in the Test and Control groups, 74.83 ± 2.51% and 75.62 ± 1.83% (*p* > 0.05), respectively; the mean values for BV/TV in the Test and Control groups were, respectively, 24.38 ± 1.83% and 23.26 ± 3.08% (*p* > 0.05); for Tb.Th, there was also no statistically significant difference (GT: 0.063 ± 0.004; CG: 0.062 ± 0.003); a similar pattern was observed for Tb.N in the Test and Control groups, with mean values of 3.91 ± 0.19 and 3.92 ± 0.25 (*p* > 0.05), respectively (Figure 5 and Figure 6).

After 60 days, in intra-thread bone microtomographic analysis, statistically significant differences (*p* < 0.05) were observed between groups for all parameters evaluated, as follows: IS/TS for CG (27.84 ± 5.41) vs. TG (58.65 ± 23.49); Po.Tot of 56.35 ± 12.05% for the Control and 71.75 ± 4.54% for the Test Group; for BV/TV, the mean values were 43.65 ± 12.05% for the Test and 28.25 ± 4.54% for the Control Group; for Tb.Th, TG (0.067 ± 0.006 mm) vs. CG (0.059 ± 0.003 mm); for Tb.N, the mean value for the TG was 6.40 ± 1.34, and 4.77 ± 0.64 for the CG. The Microtomographic Analysis of the bone external to the threads also showed statistically significant differences (*p* < 0.05) between the groups for all parameters. For Po.Tot, the averages were 66.67 ± 8.02% for Test and 76.20 ± 3.80% for Control Group; for BV/TV, the mean values were 33.33 ± 8.02% for Test and 23.80 ± 3.80% for Control Group; for Tb.Th, the averages of TG was 0.061 ± 0.003 mm and of CG was 0.058 ± 0.003 mm; for Tb.N, the mean value for Test was 5.46 ± 1.18, and 4.07 ± 0.54 for Control Group (Figure 5 and Figure 6).

### 3.2. Histomorphometric Results

#### 3.2.1. Intra-Group Analysis

**Figure 7:**
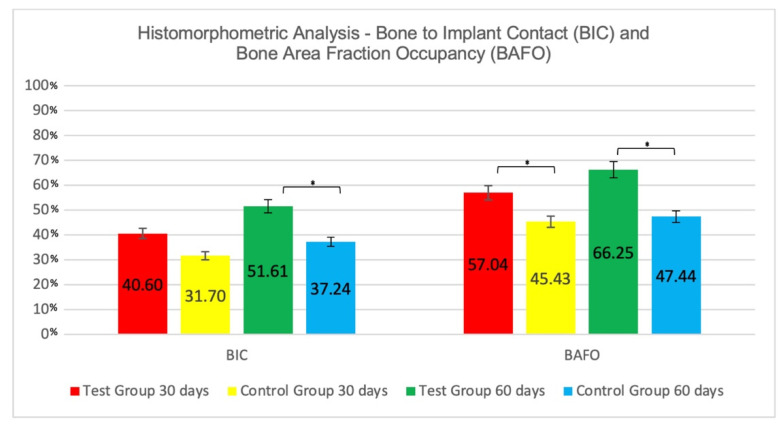
Mean values ± standard deviation percentages of Bone-to-Implant Contact (BIC) and Bone Area Fraction Occupancy (BAFO). * Statistically significant difference between groups (*p* < 0.05).

Figure 4 represents the perimeter of the implant where BIC and BAFO were evaluated. The percentage of Bone-to-Implant Contact (%) showed no significant differences between 30 days (31.70 ± 10.42) and 60 days (37.24 ± 8.57) for the CG, as well as for the TG (30 days = 40.60 ± 10.22; 60 days = 51.61 ± 13.89). There was also no significant difference for BAFO (%) between 30 (45.43 ± 3.59) and 60 days (47.44 ± 5.709) in the Control Group, and in the Test group (30 days = 57.04 ± 5.53; 60 days = 66.25 ± 11.88) (Figure 7).

#### 3.2.2. Inter-Group Analysis

At 30 days there was a numerically but not statistically significant difference on %BIC favoring the Test group (TG = 40.60 ± 10.22; CG = 31.70 ± 10.42; *p* > 0.05. At 60 days, the Test group presented higher BIC value (GT = 51.61 ± 13.89; CG = 37.24 ± 8.57), and the difference between groups was statistically significant (*p* < 0.05).

The BAFO analysis presented statistically significant differences (*p* < 0.05) between groups, favorable to the nanoHA surface, both at 30 days (TG = 57.04 ± 5.54; CG = 45.43 ± 3.60), as well as at 60 days (TG = 66.25 ± 11.88; CG = 47.44 ± 5.71) (Figure 7).

### 3.3. Analysis of Pre-Existing Cortical Bone versus Grafted Bone

After 30 days, the mean value for the BIC (%) at the pre-existing cortical bone was 92.60 ± 4.50 and 88.33 ± 5.26 for the Control and Test Groups, respectively, with no statistical difference between groups. For BAFO (%), the mean value was 93.01 ± 2.71 for the Test and 83.30 ± 7.00 for the Control groups, with a significant difference between them (*p* < 0.05). On the grafted bone area, there was a statistically significant difference (*p* < 0.05) for BIC, favoring the Test group (CG = 19.81 ± 9.82; TG = 56.00 ± 14.06). BAFO mean values presented statistically significant differences between groups (TG = 45.81 ± 8.83; CG = 22.59 ± 7.95; *p* < 0.05) (Figure 6).

After 60 days, the mean values for BIC at the cortical bone, in Control and Test groups, were 85.34 ± 8.29 and 86.01 ± 6.47 (*p* > 0.05), respectively; for BAFO, the averages were 86.92 ± 8.85 for the Test Group and 84.13 ± 6.56 for the Control Group, without a statistically significant difference between them (*p* > 0.05). In grafted bone, mean values of BIC were 43.72 ± 14.93 and 63.67 ± 10.79 for the Control and Test groups, respectively, with a statistically significant difference between groups (*p* < 0.05); for BAFO, the difference between groups was not statistically significant (TG = 57.05 ± 11.90; CG = 56.45 ± 14.59; *p* > 0.05).

**Figure 8:**
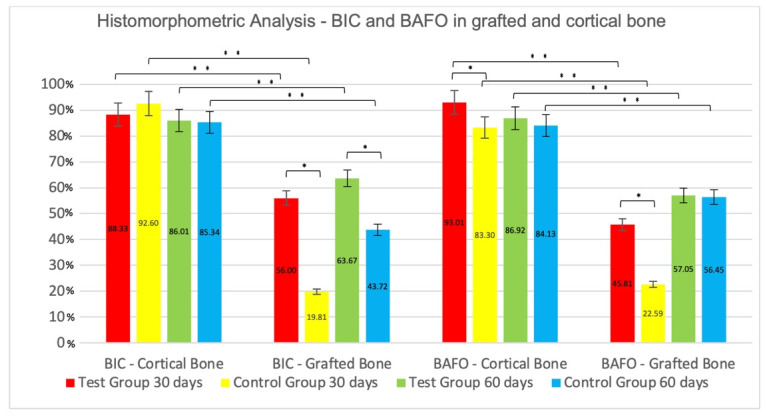
Mean values ± standard deviation percentages of Bone-to-Implant Contact (BIC) and Bone Area Fraction Occupancy (BAFO) in grafted bone and cortical bone—30 and 60 days. * Statistically significant difference inter-groups comparisons (*p* < 0.05). ** Statistically significant difference intra-groups comparisons (*p* < 0.05).

The comparison between cortical bone and grafted bone, at the same time of analysis and for the same surface (intra-group differences in relation to the type of bone), showed better and statistically significant results in favor of cortical bone, in comparison to the grafted bone (*p* < 0.05), for both implant surfaces, for all evaluated parameters and examination periods. (Figure 8).

## 4. Discussion

This in vivo study evaluated the biological performance of two different implant surfaces on sinus grafting procedures in an in vivo model by means of microtomographic and histomorphometric parameters. It was demonstrated that the nanoHA coating promoted a significant increase in bone repair, when compared to a double acid-etched implant surface, suggesting that nanoscale bioactive surface modifications may enhance bone formation in grafted areas.

Increasing implant surface roughness helps initial bone fixation, with higher values for bone–implant contact and better mechanical distribution of forces on implant surface [39]. Nanotopography influences interactions between cell surfaces, promoting higher levels of osseointegration [40]; furthermore, the deposition of bioactive molecules, such as hydroxyapatite, can additionally improve bone healing [40].

The nanoHA surface is produced with a combination of subtractive and additive methods. First, the machined implant surface is treated with double acid-etch solutions, and after that the deposition of soluble nanocrystals of HA forms a 20 nm thickness layer without detaching these crystals. The nanodeposited HA acts as a topographic agent, giving the surface medium roughness characteristics. Nanoscale HA structures help osteoblasts to synthesize mineral matrix by topographic stimuli [20] and also act as a bioactive agent, accelerating the process of osseointegration [41].

Implants with nanoHA-treated microsurface provide a more suitable arrangement, which better mimics the natural organization of bone tissue and facilitates interaction with tissue biomolecules and cell–cell communication during the healing process [16,42]. These arguments agree with the results obtained in the present study, in which the nanoHA surface presented a more organized, denser bone, with higher contact with the implant after 60 days, when compared to the DAE surface.

In the present study, bone remodeling was in a more advanced stage around implants with nanoHA-treated surfaces, especially within 60 days. It suggests that bone remodeling can occur earlier on such surfaces, despite the fact that no significant differences could be shown at 30 days between groups, which suggests that the possible effects of this surface on bone healing require a longer time in grafted areas. The absence of inter-group differences in the first 30 days can also be partly attributed to the surface of the control group. The moderately rough and microporous microtopography has inherent bioactivity through topographic action in osteoblastic cells that leads to a decreased osseointegration time [9] and better osseointegration when compared to smooth surfaces [43,44,45].

The bioactivity of nanoHA was previously related in a study comparing this implant surface to a commercially pure smooth surface, both of them installed in rabbit tibiae [18]. This biological response seems to be different according to each nanostructure used [14,23]. The present study is in accordance with these findings; nanoHA surface presented better results even with a medium roughness surface present in the Control group (in comparison to other studies that used machined surfaces). This bioactivity might have played an important role in improving osseointegration in grafted bone, mainly 60 days after implant installation. 

To the best of our knowledge, this is the first study to compare these implant surfaces on a bone-grafted area. Previous studies had tested different bone healing inducers, such as rhBMP-2 [32,33]. Thoma et al. [33] evaluated implants coated or not with rhBMP-2, and have showed a strong osteogenic reaction in the first 30 days favoring rhBMP-2, with a BIC of 32%. Yoon et al. [32] evaluated the association (TG) or not (CG) of rhBMP-2 to the deproteinized bovine mineral graft; an implant with a roughened surface was installed in the same surgery, and after 4 weeks, the authors found a BIC of 22% for TG. Although using rhBMP-2, in the implant surface or in the bone substitute, these two studies presented smaller BIC values than those found in the present study after 60 days (40%). These data suggest a high osteogenic potential of nanoHA surface, since the comparison to a well-known osteoinductive substance (rhBMP-2) showed numerically superior results of bone-to-implant contact.

When comparing microCT results of bone volume/total volume, Yoon and collaborators showed, at 30 days, numerically similar BV/TV (26.29%) to that found in the present study. Thoma and collaborators have found a decreasing trend on BV/TV over time, with values slightly higher than the present study after 30 days (27.82%) and lower than at 60 days (9.74%). This decrease was not observed in our study (30 days = 24.23%; 60 days = 43.65%). One hypothesis for such a difference is the absence of filling of the sinus cavity with a bone graft in the Thoma research. One study in monkeys [46] showed that the void occupied by the coagulum shrank substantially, when bone substitutes are not used; the Schneiderian membrane did not provide a basis for new bone formation in the early phase of healing. 

The surfaces’ performance on pre-existing cortical bone compared to the grafted bone, evaluating BIC and BAFO measurements, revealed differences in the healing pattern. Higher values were found in the cortical portion in relation to the grafted area, which corroborates a study that has shown similar results [46]. De Santis et al. [47] showed that both anorganic bovine bone granules and autologous bone grafts contributed to the healing around implants with a moderately rough surface installed immediately in elevated sinus sites in rabbits. For BIC in the cortical bone, De Santis’s study showed values of 58.9% and 60.0% for Anorganic Bovine Bone and Autologous group, respectively, after 40 days; in the present study, 30 days after surgery the values of BIC were 88.33% and 92.60% for test and control groups, respectively. These differences could be explained because the portion of implant in contact with cortical bone was machined in De Santis’s research. In grafted bone areas, at 30 days, the present study showed a BIC of 56.00% and 19.81% for Test and Control groups, respectively, and the values in 60 days rise to 63.67% and 43.72%; De Santis et al. [47] have found, 40 days after implants installation, values of 33.4% and 39.3% for Anorganic Bovine and Autologous bone, respectively. Again, the better results for the test group in the present study could be due to the different implant surfaces.

The Schneiderian mucosa does not remarkably stimulate bone formation in the early stages of healing [29]. Recently, authors have described in humans a significant difference on new bone formation as the distance to native bone increases; it seems that the inherent bone regeneration or bone growth is provided by native bone [43]. In the present study, there was an increase in percentage of BIC values in the grafted bone for both groups, from 30 (CG = 19.81; TG = 56.00) to 60 (CG = 43.73; TG = 63.67) days, with statistically significant differences (*p* < 0.05) between groups in each period of evaluation. However, the values of BIC in the cortical bone were higher for both groups when compared to the grafted bone, for all periods of evaluation. The bioactivity of nanoHA surface may have played an important role on enhancing bone formation in the grafted area, while in cortical native bone this property might not be of great importance.

## 5. Conclusions

The implant surface modified by the addition of nano-hydroxyapatite showed superior biological features compared to the double acid-etched treatment, promoting higher bone formation around the implants in an experimental model of bone repair in a grafted area, using a xenogeneic bone substitute.

## Data Availability

The data presented in this study are available on request from the corresponding author. The data are not publicly available due to ethical reasons.
